# Transactions of the Chicago Society of Physicians and Surgeons, October 12, 1874

**Published:** 1874-11-01

**Authors:** Ralph E. Starkweather


### Society Reports.

### TRANSACTIONS OF THE CHICAGO SOCIETY OF PHYSICIANS AND SURGEONS.

### REGULAR MEETING, OCTOBER 12, 1874.

*Reported by Ralph E. Starkweather, M.D.*

THE President, Dr. Bartlett, occupied the chair. After the usual preliminaries Dr. D. A. K. Steele read a report of a case of pneumatic aspiration of the pericardial sac, which appears in full elsewhere in this number of the EXAMINER.

Dr. F. H. Davis next read several interesting reports of cases.

The President reported a case of cerebro-spinal meningitis which proved fatal, in which were numerous curious features simulating hydrophobia. The discussion of this subject, including rabies, was animated and exhaustive.

Dr. Hamill reported a case of abortion with retained placenta, in which he used full doses of ergot for twelve hours, without effect. He then exhibited the fluid extract of *actea racemosa*, forty drops every two hours; the secundines were expelled in six hours; the medicine seemed to act on the body of the uterus, producing tonic contractions. He preferred it rather than ergot. He was called to the case two days after the abortion had taken place. Several members related cases much resembling the one given, with like experience, and expressed the opinion that the use of ergot in the third stage of parturition was, at least, very questionable and inexpedient.

Dr. Merriman was appointed by the President to prepare a paper to be read at the next meeting, upon the management of the third stage of labor.

The society then adjourned.

